# BIITE: A Tool to Determine HLA Class II Epitopes from T Cell ELISpot Data

**DOI:** 10.1371/journal.pcbi.1004796

**Published:** 2016-03-08

**Authors:** Lies Boelen, Patrick K. O’Neill, Kathryn J. Quigley, Catherine J. Reynolds, Bernard Maillere, John H. Robinson, Ganjana Lertmemongkolchai, Daniel M. Altmann, Rosemary J. Boyton, Becca Asquith

**Affiliations:** 1 Section of Immunology, Wright-Fleming Institute, School of Medicine, Imperial College London, London, United Kingdom; 2 Department of Biological Sciences, University of Maryland Baltimore County (UMBC), Baltimore, Maryland, United States of America; 3 Lung Immunology Group, Section of Infectious Diseases and Immunity, Hammersmith Hospital, Department of Medicine, Imperial College London, London, United Kingdom; 4 CEA-Saclay, Institute of Biology and Technologies, SIMOPRO, Labex LERMIT, Labex VRI, Gif Sur Yvette, France; 5 Institute of Cellular Medicine, Newcastle University, Newcastle upon Tyne, United Kingdom; 6 Centre for Research and Development of Medical Diagnostic Laboratories, Faculty of Associated Medical Sciences, Khon Kaen University, Khon Kaen, Thailand; 7 Department of Respiratory Medicine, Royal Brompton & Harefield NHS Foundation Trust, Sydney Street, London, United Kingdom; La Jolla Institute for Allergy and Immunology, UNITED STATES

## Abstract

Activation of CD4^+^ T cells requires the recognition of peptides that are presented by HLA class II molecules and can be assessed experimentally using the ELISpot assay. However, even given an individual’s HLA class II genotype, identifying which class II molecule is responsible for a positive ELISpot response to a given peptide is not trivial. The two main difficulties are the number of HLA class II molecules that can potentially be formed in a single individual (3–14) and the lack of clear peptide binding motifs for class II molecules. Here, we present a Bayesian framework to interpret ELISpot data (BIITE: Bayesian Immunogenicity Inference Tool for ELISpot); specifically BIITE identifies which HLA-II:peptide combination(s) are immunogenic based on cohort ELISpot data. We apply BIITE to two ELISpot datasets and explore the expected performance using simulations. We show this method can reach high accuracies, depending on the cohort size and the success rate of the ELISpot assay within the cohort.

## Author Summary

When studying the host immune response, a central question is: “which peptides elicit CD4^+^ T cell responses?” ELISpot assays are used to assess if subjects have responded to a given peptide. However, to determine which of the HLA-II molecules coded by the host HLA genotype is responsible for the reaction requires additional analysis. We present a Bayesian approach to solve this problem and have implemented it for use with the statistical language R under the BIITE moniker. Importantly, the aim of BIITE is to interpret experimental data, not to make *in silico* predictions. The method considers the immunogenicity of all HLA (in a cohort of patients) with respect to a given peptide simultaneously, in order to deal with linkage disequilibrium between genes of the HLA locus. Furthermore, users can enter additional information they might have (from literature or other experiments) in the form of prior information. The method is not exclusive to the HLA genes and can be used to attribute positive binary outcomes to any multi-allelic set of genes.

This is a *PLOS Computational Biology* Methods paper.

## Introduction

Adaptive immunity relies on the recognition of non-self peptides by T cell receptors to mount an effective response against an infection. Both self and non-self peptides are presented on the cell surface by human leucocyte antigen complex (HLA) molecules using two pathways: the HLA class I pathway presents cytosolic peptides on HLA class I molecules to CD8^+^ T cells, while the HLA class II pathway presents peptides of proteins that have been internalized by antigen presenting cells (APCs) to CD4^+^ T cells.

Identifying which peptide:HLA (pHLA) complexes give rise to an immune response in any infectious, autoimmune, allergic or oncogenic disease is paramount to understanding host defence and pathogenesis and informing epitope vaccine design. Furthermore, the ability to define the specific pHLA complexes dominating a given response is central to both the theoretical understanding of HLA-disease associations and to the practicalities of designing experiments that use pHLA tetramers to enumerate and characterise specific T cells. Only a small proportion of HLA-binding peptides are able to elicit a T cell response in the context of a given HLA class I [1] or class II molecule; identifying which pHLA complexes are immunogenic is not trivial.

The most widely used experimental method for investigating immunogenicity in humans is the ELISpot assay [2]. Interpreting ELISpot responses of CD8^+^ T cells is relatively straightforward. Given an individual’s HLA class I genotype there are a limited number of HLA-I molecules that could be present (a maximum of 6); this together with the highly specific nature of the HLA class I peptide binding register means that identifying which HLA molecule is responsible for a positive response in a given individual to a certain peptide is usually unambiguous.

Here we focus on the more demanding problem of defining immunogenic pHLA-II complexes. That is, having established that an individual of known HLA class II genotype gives a positive CD4^+^ T cell ELISpot response to a given peptide, how could one infer which of the class II molecules was responsible for eliciting this response? Multiple factors complicate this analysis. Firstly, HLA-II molecules are heterodimers whose alpha and beta chains are encoded at separate genetic loci. Given their high polymorphism (the exception being the locus encoding the DR alpha chain) and the fact that their products might be paired either *in cis* or *in trans*, the number of expressed DP and DQ molecules in heterozygotes is theoretically doubled [3–6]. Furthermore, the DR alpha chain can be paired with the DQ beta chains [7]. Secondly, the DR beta chain can be sourced from between 2 to 4 loci depending on the subject. All humans possess two copies of the *DRB1* locus. These can be complemented by a maximum of two of *DRB3*, *DRB4* or *DRB5* (one per chromosome). Consequently a maximally heterozygous individual may have 14 distinct HLA class II molecules. Thirdly, expression levels seem to differ [8] between different chains, leading to differential presentation of HLA-II molecules on the cell surface. Fourthly, (as for the class I genes), the genes of the HLA-II locus are in strong linkage disequilibrium, complicating the attribution of T cell responses to specific HLA-II loci. Lastly, the class II peptide binding grove is open at both ends and so it can accommodate peptides of variable length. This means that several amino acids in a given peptide could be anchor residues, complicating the *in silico* scanning of peptides for binding motifs. Together these factors mean that identifying which of an individual’s 3–14 possible HLA class II molecules is responsible for eliciting a positive CD4^+^ T cell response is problematic.

Historically, this problem has been addressed by cloning T cells and dissecting responses functionally, for example with HLA transfectant APC panels. However, this is intractable for high-throughput epitope mapping studies. While methods exist for predicting binding of peptides to HLA class II molecules, for example NETMHCIIpan [9], our aim is different in two important respects. Firstly, we are seeking a method to interpret experimental data rather than to make *in silico* predictions; secondly, we aim to infer immunogenicity rather than peptide binding. Paul et al. have recently described the RATE method [10] which addresses the same question. Their method calculates the relative frequency (RF) of positive CD4^+^ T cell ELISpot outcomes from multiple individuals in the HLA+ and HLA- groups in order to discover immunogenic pHLA combinations. In contrast, we propose a Bayesian framework to determine the immunogenicity of peptide:HLA-II complexes for a given peptide, which allows us to consider all HLAs simultaneously. We have implemented this in the R package BIITE (Bayesian Immunogenicity Inference Tool for ELISpot).

## Methods

### Model

We will use the abbreviation HLA to denote HLA-II, but the same approach could be used to determine HLA class I peptides from CD8^+^ T cell ELISpot data. Assume we have ELISpot data *D* for a single peptide in a cohort of *N* individuals, in which a total of *m* HLA molecules are present. We wish to obtain the peptide:HLA immunogenicity, *E*, for each of the *m* HLAs as a number between 0 and 1; this is approximately the probability that a pHLA combination results in a positive ELISpot in a randomly chosen individual (with the relevant HLA allele) and would be exact if each subject presented exactly one HLA. Hence, the hypothesis space we will explore is [0, 1]^*m*^. Bayes’ theorem (see S1 Text for more information) states that the posterior likelihood *P*(*H*|*D*) for a hypothesis *H* = (*E*_1_,*E*_2_,…,*E*_*m*_) ∈ [0,1]^*m*^ is proportional to the product of the prior *P*(*H*) and the likelihood *P*(*D*|*H*):

P(H|D)∝P(D|H)P(H).

### Likelihood

We define the likelihood *P*(*D*|*H*) multiplicatively:

$$P\left(D|H\right)=\prod_{i=1}^{N}P\left(D_{i}|H\right),$$

where *D*_*i*_ denotes the data for one individual and *P*(*D*_*i*_|*H*) is defined as

$$P\left(D_{i}|H\right)=\;\{\begin{array}{rr} \prod_{j=1}^{m}{\left(1-E_{j}\right)}^{n_{ij}} & \text{if subject }i\text{ has a negative ELISpot,}\\ 1-\prod_{j=1}^{m}{\left(1-E_{j}\right)}^{n_{ij}} & \text{if subject }i\text{ has a positive ELISpot}. \end{array}$$ (1)

Here, *n*_*ij*_ is the copy number of HLA allele *j* in subject *i*. The logic behind this formula is explained in more detail in S1 Text.

### Prior

The algorithm allows for the inclusion of pre-existing knowledge or beliefs. These could be results from previous experimental assays or from prediction algorithms. When no prior knowledge is given, the prior is the uniform distribution on [0,1]^*m*^. When prior knowledge is available, we assume it is on an HLA molecule-by-molecule basis:

$$P\left(H=\left(E_{1},E_{2},\ldots ,E_{m}\right)\right)=\;\prod_{j=1}^{m}P\left(E_{j}\right).$$

In our analysis, we used prior data from NetMHCIIPan [9]. NetMHCIIPan was used to predict the binding of peptide:HLA combinations with a cut-off of IC = 500nM. The combinations below this threshold were assigned a Beta prior with mode 0.35 and SD 0.2; combinations above this threshold were assigned a Beta prior with mode 0.001 and SD 0.15 (see S1 Fig).

### Implementation

In order to find the posterior density *P*(*H*|*D*), we implemented the Metropolis-Hastings algorithm [11], which is explained in more detail in S1 Text, to construct Markov chains of length 100,000, as these were found to return the same marginal distributions as chains of length 10^6. The model was implemented in R version 3.1 [12]. The graphical output was generated with the R package ggplot2 [13]. The code is available on http://github.com/liesb/BIITE as an R package. It can be installed using the R library devtools. In R, use the command install.packages("devtools") to install devtools. Then, to install BIITE, load devtools using the command library(devtools); next, use devtools::install_github("liesb/BIITE") to retrieve BIITE from its github repository. To load the package, use library(BIITE).

### Input format

An example input file can be found in S4 Table. Each row in the file represents a subject. There should be a column for each HLA in the population, which contains the subjects’ copy number of that gene. For each peptide that is tested, there is a column, containing a logical value: TRUE if the subject delivered a positive ELISpot for the peptide, otherwise FALSE. The order of the columns does not matter, although preferably the ELISpots columns and the HLA columns form one block each. The HLA data can be entered in two-digit or four-digit format or a mixture of the two (see the example in S1 Table): most HLAs are defined on the two-digit level, but DRB1*15 has been split into DRB1*15:*01* and DRB1*15:*02*). Since ‘*’ is a special character in the R language, it should not be used in the column names and can be replaced by an underscore.

### Interpretation of the posterior

In order to estimate the immunogenicity of each peptide:HLA combination, we consider the marginal distributions *P*(…,*E*_*j*_,…|*D*) for each of the HLA molecules in the population. If the data contain little information about the immunogenicity of a pHLA combination (e.g. the HLA molecule is present only at very low frequencies in the population), then this marginal posterior distribution will not differ very much from the prior it was assigned.

If a pHLA combination has a relatively flat posterior distribution then its immunogenicity cannot be reliably determined; this will occur when limited information about the pHLA is contained in the data. We eliminate these unreliable combinations by only considering pHLA combinations which differ from the uniform distribution. We quantify the difference between the posterior and the uniform using the Kullback-Leibler divergence *D*_*KL*_(*P*(*E*_*j*_|*D*)||Unif(0,1)). If this difference is smaller than *D*_*KL*_(Beta(2,1)||Unif(0,1)) we say there is insufficient information to determine the immunogenicity. We have chosen the threshold to be determined by the Beta(2,1) distribution, which naturally arises as the posterior distribution of the parameter of the binomial distribution after one (positive or negative) observation, when assuming a uniform prior. In other words, if we want to know what the probability *p* is of a coin landing heads in a toss, and we are only allowed one experiment with no prior information, Beta(2,1) (or Beta(2,1)) is the best description of *p*. Hence, requiring that the Kullback-Leibler divergence passes the aforementioned threshold, is asking that the posterior contains more information that we would get from a single coin toss. This threshold is arbitrary and can be adjusted by the user.

To obtain a single estimate for *E*_*j*_, we propose using the mode of the marginal posterior distribution, but other summary statistics could be considered. In our analyses, using either the mean or median of the marginal posterior distribution yielded very similar results. These three summary statistics are all provided in the output of the algorithm (see S8 Table for an example where we have retained only the mode). Our interest lies in determining, for a given peptide, which HLA molecules were responsible for the observed positive ELISpot assays in the dataset, i.e., which HLA has the highest explanatory power. This can be done as follows. Based on the output file, we can rank the HLAs from highest posterior mode to lowest posterior mode; the HLAs ranked highest are the ones identified by BIITE to be the most immunogenic. Next, we turn to the subject data and determine which of their HLAs has the highest rank. For a subject with a positive ELISpot, this one ‘highest ranked HLA’ is considered to be the HLA responsible for the positive ELISpot result. To check whether a single HLA has high explanatory power, we determine how many subjects with a positive ELISpot carry that HLA, and in how many of these subjects the HLA is the highest ranked HLA.

### Worked example

To illustrate the application of the method we have created a worked example. The input file is provided in S4 Table, the corresponding output file (for pep_1) in S8 Table. The HLAs are ranked from high to low (‘Posterior mode’ column). For each HLA, we count how many subjects carry the HLA and have produced a positive ELISpot (‘# Carriers with positive ELISpot’). For example, of the 73 carriers of DQB*03, 67 had a positive ELISpot. Since DQB*03 is also the highest ranked HLA overall, all of these 67 positive ELISpots are ‘explained’ by DQB*03. For the second-highest ranked HLA, DRB1*14, 25 of 28 subjects had a positive ELISpot. Fourteen of those 25 are explained by DRB1*14 (the other 11 DRB1*14 carriers with a positive ELISpot also carry the higher ranked DQB*03, which ‘explains’ their positive ELISpot). In contrast, the lowest-ranked HLA, DQB*02, has 54 carriers that produced a positive ELISpot, but none of these positive results are explained by DQB*02.

### Validation on a *Burkholderia Pseudomallei* dataset

The bacterium *Burkholderia Pseudomallei* is the causative agent of melioidosis. PBMCs from a small cohort (N = 38) of exposed sero-positive blood donors were used to test 17 overlapping 20-mers of the alkyl hydroperoxide reductase protein (AhpC, BPSL2096) by IFNγ ELISpot assay [14]. Results that were 2 standard deviations (SD) above the mean of medium only control were considered positive.

Each subject was HLA genotyped at the *HLA-DRB1* and *HLA-DQB1* loci. The observed alleles and their frequencies in the cohort are listed in S1 Table.

We applied the algorithm to each of the 17 peptides separately, both with and without the inclusion of prior data.

We used two datasets for validation. In the first dataset, immunogenicity of pHLA complexes for 6 HLAs (DRB1*01, DRB1*04, DRB1*15:*01*, DRB1*15:*02*, DQB1*06, DQB1*08) was determined using transgenic mouse models that express a single (human) HLA-II molecule (and no mouse MHC class II) and were challenged with the 17 different peptides in turn; IFNγ ELISpot was then used to determine whether peptides were immunogenic (number of SFCs larger than mean of SFCs numbers in medium-only + 2SD) [14]. The second dataset consisted of relative peptide binding affinities for all 17 overlapping 20-mers of the protein with respect to 10 HLA-II molecules; this was evaluated with competitive ELISA using an automated workstation. A peptide was considered to be binding to the HLA molecule when the relative binding affinity was less than 100 [14].

The immunogenicity of each pHLA combination was calculated as the mode of the marginal posterior distribution. We constructed ROC curves to compare these values to the pHLA binding data and transgenic immunogenicity data.

### Validation on a *Pseudomonas Aeruginosa* dataset

The bacterium *Pseudomonas Aeruginosa* (PA) is an environmental organism that can cause infection in damaged lung or the immune compromised host. PBMCs from a small cohort (N = 58) of patients with a chronic lung disease associated with recurrent lung infection and progressive lung damage, called bronchiectasis, were used to test 34 overlapping 20-mers of the OprF protein using IFNγ ELISpot assays [15]. We used a cut-off of 2SD above the mean of medium only control to decide whether a particular ELISpot result for an individual was positive or negative.

Analogously to the Burkholderia dataset, we had access to a immunogenicity data derived from transgenic mouse models and binding data from peptide-binding assays [15]. Transgenic mouse models were available for DRB1*01, DRB1*04 and DRB1*15; binding data was available for 7 *HLA-DRB1* alleles.

Each subject was HLA-typed at the *HLA-DRB1* and *HLA-DQB1* loci. The observed alleles and their frequencies in the cohort are listed in S2 Table. Each subject is classified into one of two groups, based on the results of sputum microscopy and culture by standard microbiological techniques. Individuals in group 1 were never sputum culture positive for PA, while individuals in group 2 were sometimes or frequently sputum culture positive for PA over a period of 6 months.

We first applied the algorithm to each of the 34 peptides separately, both with and without the inclusion of prior data, including all subjects. Next, we applied the algorithm to each of the two groups (as described above) separately.

### Validation on synthetic datasets

In order to assess the validity of the method and the effect of sample size on the outcomes of the algorithm, we resampled (with replacement) HLA class II haplotypes from the Burkholderia dataset (n = 10, 30, 50, 70, 100, 150, 200). We randomly assigned each peptide:HLA molecule an immunogenicity value *E*_*j*_∈[0,0.55]; whether a peptide:HLA combination was considered immunogenic was decided by a cut-off on *E*_*j*_; we considered a range of different cut-offs (0.1, 0.2, 0.3, 0.4, 0.5). We then simulated ELISpot data in the following way: for each subject, the probability of having a positive ELISpot was calculated using eq (1) above, whether the individual had a positive or negative ELISpot was then decided by drawing a random number from the unit interval and comparing it to this probability; if the random number was above the computed probability then the individual had a negative ELISpot, if it was below they had a positive ELISpot. We constructed ROC curves and calculated the AUC for the different cut-offs listed above.

### Comparison to RATE

Recently, Paul et al [10] have proposed a novel algorithm “RATE” to determine immunogenic peptide:HLA complexes from ELISpot data. We applied the RATE tool to both the *Burkholderia* and the *Pseudomonas* dataset. RATE returns two reports: a concise one, which contains the pHLA combinations who have a positive ELISpot relative frequency (RF) greater than two; and a complete report which ranks all peptide:HLA combinations in the population. We calculated the AUC for each dataset based on the RF of all the pHLA complexes whose posterior distributions passed the Beta(1,2) cut-off criterion described above.

### Validation on an HIV-1 dataset (HLA class I restricted CD8+ T cells)

While BIITE was designed to address the issue of elucidating peptide:HLA immunogenicity for class II HLA, we hypothesized that it could solve a larger class of problems (see Discussion). To explore the performance of BIITE on different datasets, we downloaded patient data and CD8^+^ T cell ELISpot outcomes for a range of HIV-1 peptides from [16]. We used 2-digit level typing for all HLAs, except for HLA-B*15 (where we used B*15:*03* and B*15:*10* if this subtyping was provided, and B*15 else), HLA-B*58 (B*58:*01* and B*58:*02*) and HLA-A*30 (A*30:*01* and A*30:*02*). For other HLA alleles, we either lacked sufficient HLA-typing on the 4-digit level, or there was one dominant 4-digit allele; see S5 Table for an overview. We applied the algorithm to the 32 peptides used for validation in [10] to sample the posterior probability distribution on the hypothesis space (sample size 250,000). The output was interpreted as described above and peptide:HLA combinations that were predicted to be immunogenic were matched against entries in the LANL A-list of HIV-1 epitopes and the general LANL database.

## Results

**Fig 1:**
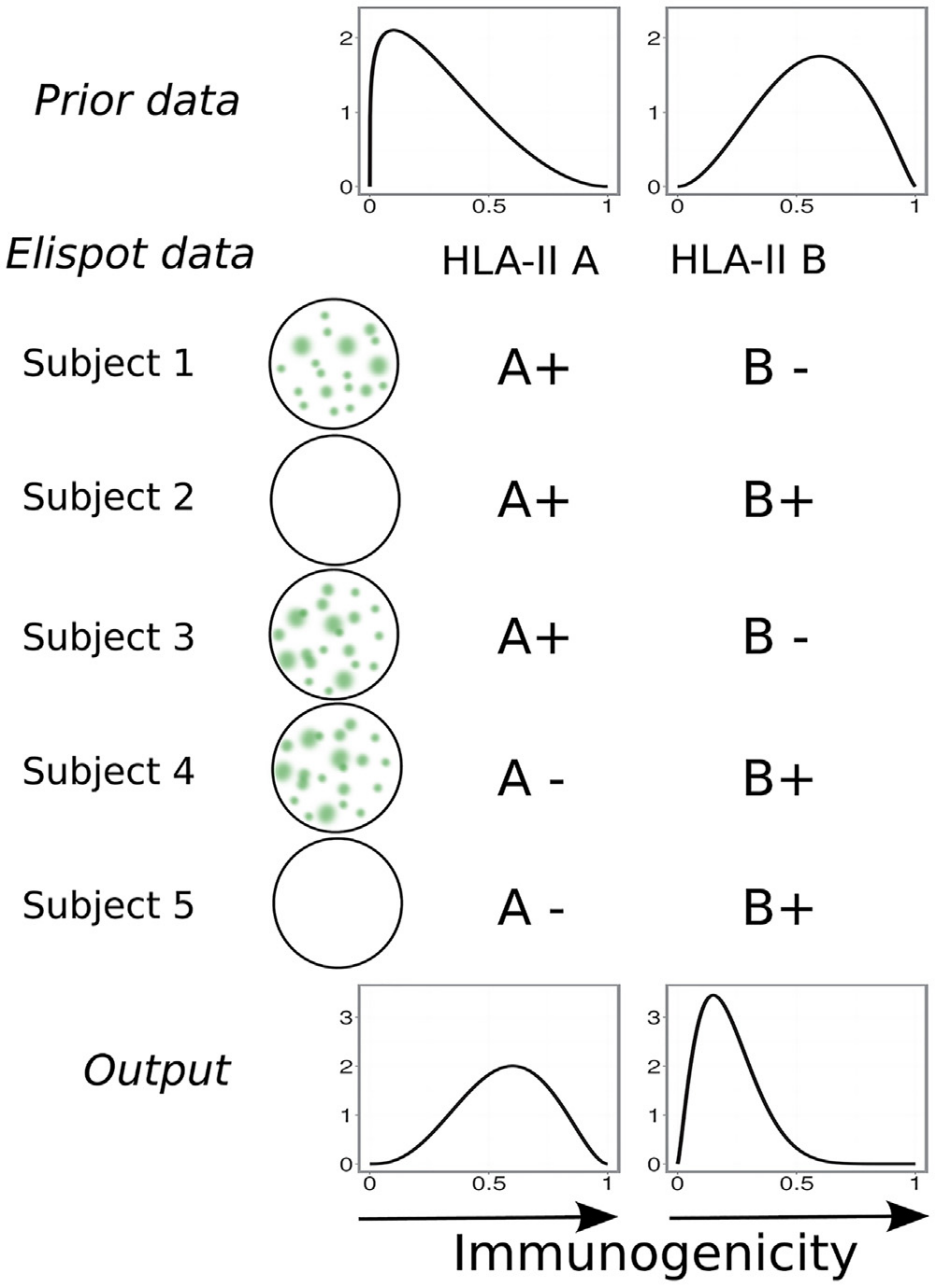
Outline of the algorithm. We aim to describe the immunogenicity of a peptide:HLA combination as a number between 0 and 1. For each subject, the outcome of the ELISpot assay is known, together with a list of their HLA alleles. The user can add prior knowledge in the form of a prior distribution. In the example, the prior knowledge describes the belief that HLA class II allele *A* is not immunogenetic in combination with the peptide, while HLA class-II allele *B* is. The output of the algorithm consists of posterior marginal distributions of the values *E*_*j*_. In the example, it is found that allele *A* is more immunogenic that allele *B* in combination with the peptide.

We designed and implemented a Bayesian framework, BIITE, to determine peptide:HLA immunogenicity from ELISpot data; Fig 1 outlines the algorithm. The algorithm determines the probability density function of the immunogenicity. The immunogenicity of a pHLA combination is the approximate probability that the peptide results in a positive ELISpot in a randomly chosen individual with the relevant HLA allele (this value would be exact if each subject expressed only one HLA). Strictly necessary inputs are the ELISpot experiment outcomes for each subject, together with their genotype for the loci of interest. Optionally, the user can also provide prior information to the algorithm; this is done on an HLA molecule-per-molecule basis. The output of the algorithm is a sample of the posterior distribution *P*(*H*|*D*), which we visualize by plotting the marginal posterior density for each peptide:HLA combination. This can be interpreted as the probability density function of the immunogenicity.

### Validation on *Burkholderia* dataset

In order to test the BIITE algorithm, we used CD4^+^ T cell ELISpot data from a small cohort of exposed sero-positive individuals (N = 38). All overlapping 20-mer peptides from the BPSL2096 protein were tested (see Methods).

**Fig 2:**
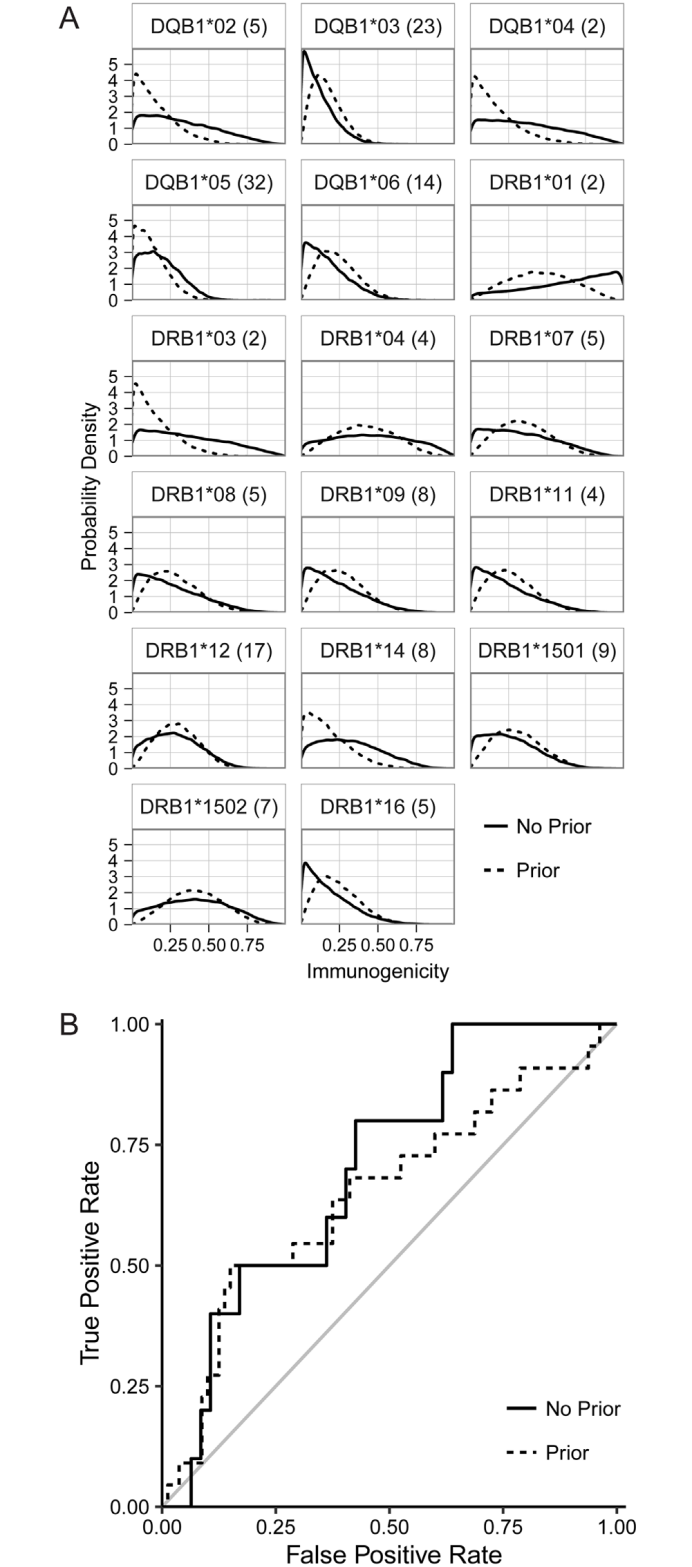
Application of the algorithm to the *Burkholderia* data set, with (dashed lines) and without (solid lines) inclusion of prior information. (A) Marginal posteriors of the peptide:HLA immunogenicities for BPSL2096 1–20 (Allele count in the cohort in brackets). (B) ROC curves were generated by comparing the mode of the posterior distributions against the transgenic mouse data set. For these curves, we have removed all posterior distributions whose Kullback-Leibler divergence with respect to the uniform distribution on the unit interval was smaller than that of the Beta(1,2) distribution.

In Fig 2A, we present the marginal posterior distributions of the immunogenicity (solid lines) for all HLA molecules in combination with a single, representative peptide (BPSL2096, 1–20), calculated using a uniform prior. Especially for rare alleles (see S1 Table), the distributions are rather flat and uninformative. We also show the marginal posterior distributions, now assuming a non-uniform prior as described above (Fig 2A, dashed lines). The posterior distributions are now markedly more pronounced. This is of course driven by the prior assumptions, especially for the rare alleles. Nevertheless, there are divergences between the posterior and the prior. For example, for the representative peptide shown in Fig 2A both DRB1*01 (2 carriers) and DRB1*08 (4 carriers, one of which is homozygous), were assigned a positive prior (i.e. both were predicted to bind the peptide), yet they have different posteriors, reflecting the fact that both DRB1*01 carriers had positive ELISpot results, whilst there were two DRB1*08 carriers with a negative ELISpot.

We compared the posterior marginal distributions to the transgenic mouse model data (6 data points for each of the 17 peptides). To do so, one needs to collapse the posterior distribution to a decision: is the pHLA combination immunogenic or not, according to the model? For this analysis, we used the mode of the marginal posterior distribution as a summary statistic, and the decision rule was based on the mode being above a certain threshold. We then constructed a ROC curve by varying this threshold (Fig 2B). To interpret the strength of an algorithm, one compares it to the diagonal; this is the theoretical ROC curve for a random decision rule. The observed AUC is 71.3% when we use a uniform prior, and 65.4% when using a prior based on the predictions of NetMHCIIPan.

### Validation on *Pseudomonas* dataset

**Fig 3:**
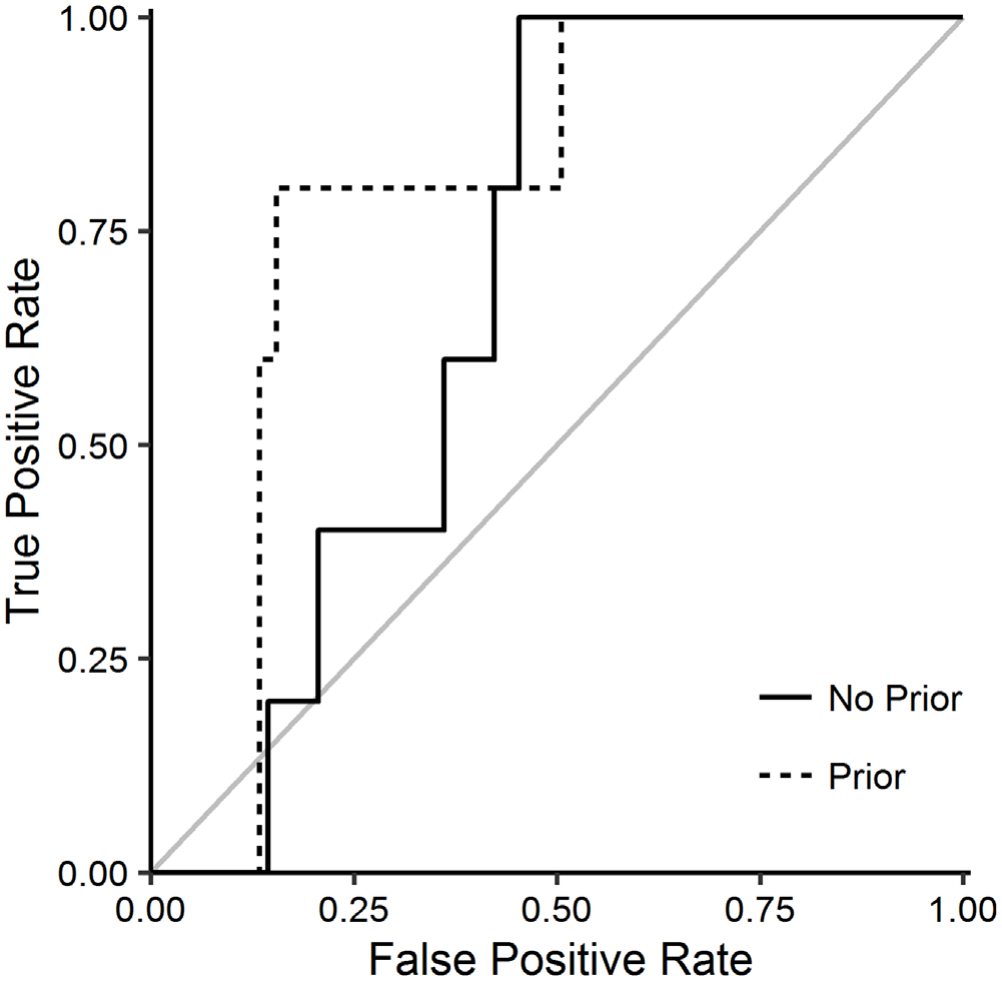
ROC curves for the analysis of the *Pseudomonas* data set, with (dashed line) and without (solid line) inclusion of a predictive prior.

We performed an additional analysis on a *Pseudomonas* dataset, comprised of N = 58 subjects. The AUCs we found were 68.8% (without predictive prior), 79.3% (with predictive prior) (see Fig 3).

The subjects in the *Pseudomonas* study were divided into two groups, based on the number of positive sputum tests they had delivered. In this study, individuals with a high number of sputum cultures positive for PA had a lower immune responsiveness (lower number of peptide that elicited a T cell response) compared to individuals that were never sputum culture positive for PA, indicating that there were two different clinical groups with different peptide immunogenicities. Hence, we applied our algorithm to each of the groups separately, and predicted that the algorithm would perform poorly on the data from group 2 (who had very low response rates in their ELISpot assays). Indeed, for group 2 (subjects with at least one positive sputum sample), we found that BIITE was unsuccessful at resolving the HLA:epitope combinations found to be immunogenic in transgenic mice (AUC: 32% without prior; 69% with prior); this apparently low performance is not unexpected as these combinations were not immunogenic in humans. For group 1, BIITE reached AUCs of 69% (without prior) and 73% (with prior).

### Determinants of performance

#### Cohort size

**Fig 4:**
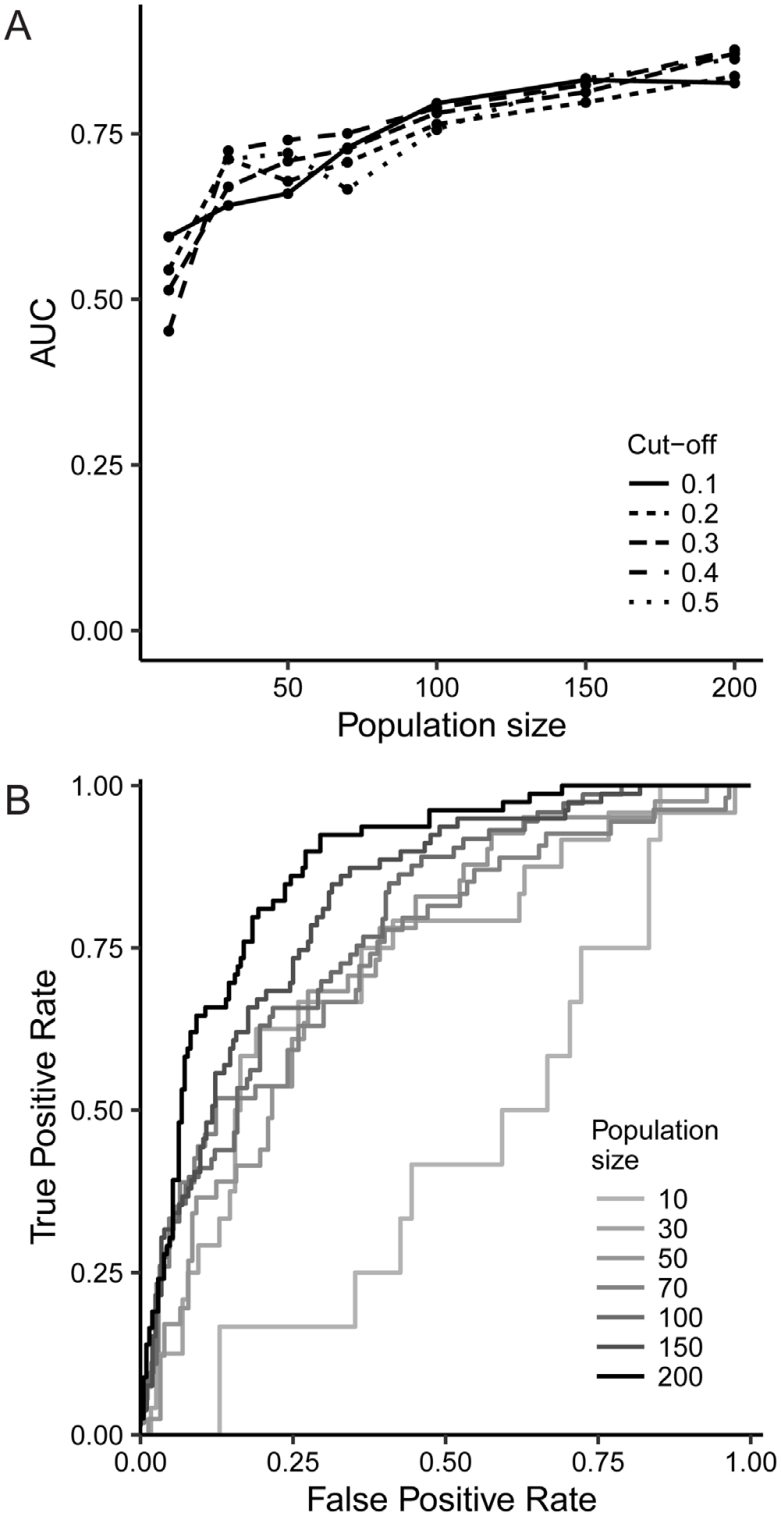
Simulated data based on the *Burkholderia* dataset. (A) Evolution of the AUC with sample size, for different cut-offs on what is considered to be an immunogenic response. (B) ROC curves for different population sizes, based on a cut-off for immunogenicity of 0.4.

To test the effect of the sample size of the ELISpot dataset, we modelled 17 *in silico* peptides based on a population of 200 subjects whose HLA class II haplotype was drawn (with replacement) from the *Burkholderia* cohort. We set peptide immunogenicity such that the odds ratio of having a positive ELISpot was similar to that in the *Burkholderia* dataset (S2 Fig). We then applied the algorithm to this new population and to subpopulations thereof. To validate the performance of the model, one needs to first decide which of the pHLA combinations are considered immunogenic. We did so by choosing a cut-off on the immunogenicity value *E*_*j*_ of each simulated pHLA combination; different cut-offs were considered (see Methods). For each of these cut-offs, we construct ROC curves and compute the AUC. Fig 4A shows the values of the AUC as a function of the cut-off on *E*_*j*_ and the population size. Unsurprisingly, performance of the model as measured by AUC increases with population size. Optimal AUCs were reached when the cut-off was taken at 0.4. Fig 4B shows the ROC curves associated with this cut-off. It is interesting to note that in our original *Burkholderia* dataset, where sample size was 38, we reached an AUC of 71%, which is comparable to the results found for sample sizes 30 and 50 here (72% and 75% respectively).

#### Crossreactivity

We also investigated how the crossreactivity of the peptide affected the performance of the BIITE algorithm. Intuitively, we expect that is it easier to determine the HLA ligands of a peptide that is poorly reactive (ie, not many positive ELISpots). This is easiest to understand in the extreme case of immunogenicity values *E*_*j*_∈{0,1}: each negative ELISpot would indicate that none of the HLAs of the subject is immunogenic; while a positive ELISpot result only informs us that (at least) one of the subject’s HLAs is immunogenic in combination with the peptide. In this extreme case, when all the subjects produce positive ELISpots, it is impossible to attribute these positive outcomes to any HLA.

To confirm the effect of the number of positive ELISpots, we ordered the peptides in the *Burkholderia* dataset based on the success rate (i.e. how many positive results they produced) in the ELISpot assay in our cohort, and grouped the peptides accordingly (see S3A Fig). We can now compute the performance of the algorithm on each of these subsets. Following the reasoning above, we predict that the AUC will be negatively correlated with the success rate (S3B Fig); this is indeed the case (S3C Fig). Note that if very few positive ELISpots are observed, the algorithm might also underperform: in the extreme case of observing one positive ELISpot, there are many candidate HLAs in that subject that could be presenting the peptide. If these HLA molecules are present at similar frequencies in the cohort, the algorithm is unable to distinguish which of the possible pHLA complexes is responsible for triggering the CD4^+^ T cell response.

#### Comparison to RATE

To our knowledge, there exists only one other algorithm, RATE [10], which uses ELISpot data to infer immunogenicity of pHLA complexes. In contrast with our Bayesian approach (which allows for inclusion of prior data and considers all HLAs simultaneously), the RATE software uses the frequentist exact Fisher test to compare the odds ratios (and the relative frequency, RF) of having a positive ELISpot in the HLA+ and HLA- groups. We first applied RATE to the *Burkholderia* dataset; it returned two potential pHLA restrictions with high RF: peptide5:DRB1*01 and peptide5:DQB1*04. Transgenic mouse data is only available for the first combination and was negative. Both combinations have a high (>0.9) posterior mode in our analysis, but lack power (Kullback-Leibler divergence below Beta cut-off) as both occur only twice in our cohort. The overall AUC based on RF was 53.6% (compared to 71.3% for BIITE). When applied to the *Pseudomonas* dataset, RATE returned 54 pHLA combinations with RF greater than 2; unfortunately, there is no transgenic mouse data available for any of these combinations. Based on the pHLA combinations that were tested in transgenic mice, we found that RATE reached an AUC of 49% (compared to 68.8% for BIITE). In the two subcohorts of the *Pseudomonas* dataset, RATE reached AUCs of 55% in group 1 (compared to 69% or 73% for BIITE, depending on the inclusion of a prior based on binding predictions, see above) and 29% in group 2 (compared to 32% or 69% for BIITE).

#### Comparison to NetMHCIIPan

Immunogenicity and binding affinity are correlated and so it is also instructive to compare our algorithm with the best available affinity predictor, namely NetMHCIIPan [9,17]. We used NetMHCIIPan to construct ROC curves, based on the binding threshold, for the pHLA combinations we had previously considered. For the *Burkholderia* data, this yielded an AUC of 67.7% (compared to 71.3% from our analysis). Since we also obtained experimental binding data for several pHLA combinations from the *Burkholderia* dataset, we were interested in the cases where the binding assay and transgenic mouse models gave discordant results. These can be divided in two groups: one where the transgenic mouse model suggests immunogenicity, while the binding assay indicates there is no stable binding, and vice versa. In the first group there were five such observations. Only one of these (20%) is predicted to bind by NetMHCIIPan. Our algorithm is powered to determine 3 of the pHLA combinations of which two (66%) are correctly identified as immunogenic (S3A Table). The second group, which contains pHLA combinations which reach stable binding but are not immunogenic based on the transgenic mouse study, is unsurprisingly larger, with 34 such combinations. Of these, NetMHCIIPan predicts 11 (32%) to be binding (at the 500nM cut-off, see S3B Table); this represents a relatively low success rate for predicting binding but would be a high failure rate for predicting immunogenicity as none of these pHLA are immunogenic. As for our analysis, 16 combinations were informative (reached the Kullback-Leibler cut-off, S3C Table); of these, two (12.5%) were incorrectly predicted to be immunogenic. This indicates that BIITE can successfully distinguish between binding and immunogenicity. Another important benefit of BIITE compared to NetMHCIIPan is that the results of BIITE are cohort-specific. That is, immunogenicity in two cohorts (e.g. controllers and non-controllers) infected with the same pathogen can be compared using BIITE. BIITE may find the same HLA:peptide combination to be immunogenic in one cohort but not in another allowing an analysis of differing immune control between the cohorts whereas NetMHCIIPan can only be used to determine whether a particular HLA:peptide combination is ever immunogenic. This is illustrated by our work on the PA dataset. In that dataset, we could distinguish clinically between two groups which were marked by different ELISpot response rates (group 2 i.e. “non-controllers” were poorly responsive with few positive ELISpots). Since NetMHCIIPan does not use cohort data, it produces the same peptide:HLA binding predictions (which in turn serve as a proxy for immunogenicity) for both groups and hence fails to capture the real differences in immunogenicity between the two groups. If we use transgenic mouse data as the validation dataset for the poorly responsive group 2 then NetMHCIIPan reached an AUC of 81.3%, compared to BIITE’s AUC of 68.8% (S4 Fig); however this analysis is misleading. The cohort produces very few positive ELISpot responses and, accordingly, BIITE identifies very few HLA:peptide combinations as being immunogenic compared to NetMHCIIPan. Arguably, BIITE’s prediction of very few immunogenic peptide:HLA combinations compared to NetMHCIIPan is a more accurate representation of immunogenicity in this poorly responsive group.

#### Effect of HLA characteristics on model outcomes

Two concerns might arise regarding the performance of our model: firstly, the model might prefer to assign higher immunogenicity values to HLAs which are more common, as they might ‘hide’ less common but immunogenic alleles in their hosts. Secondly, we want to ensure our analysis goes beyond a simple ‘counting’ algorithm, in which one considers immunogenic exactly those pHLA combinations for which the highest percentage of HLA carriers produce positive ELISpots. In order to confirm that the model is able to detect immunogenicity of peptide:HLA combinations where the HLA is rare in the population, we plotted the predicted immunogenicity (posterior mode) of each pHLA combination in the Burkholderia data set against the number of carriers of the HLA. While having a low number of HLA carriers is restrictive in the sense that their posterior distributions do not reach the Kullback-Leibler cut-off, those that do pass this requirement can be found to be immunogenic by the algorithm (S5A Fig). Similarly, the percentage of positive ELISpots per HLA allele is not strongly correlated with the posterior mode (S5B Fig); if we simply use the count to predict immunogenicity then the AUC is very low (30%) suggesting our algorithm is contributing far more than simply counting.

### Validation on an HIV-1 dataset

As described in the methods, we also analysed the HLA-I dataset that was analysed in [10]. For each peptide, HLAs were ranked by descending posterior mode provided by BIITE. To validate the results, we compared the outcome to the LANL A-list [18] of best defined epitopes in HIV-1. This is not ideal, since a peptide:HLA combination can be immunogenic without being featured on this A-list (i.e. we will tend to overestimate false positives); nevertheless, we used this list to determine lower bounds on the AUC. Both BIITE (AUC = 81%) and RATE (AUC = 77%) performed well and were able to identify A-list peptide:HLA combinations (S6 Table). However, RATE’s ‘concise output file’ (S7 Table) included a number of peptide:HLA combinations which are most likely not immunogenic but appear to be so due to linkage in the *HLA* locus (highlighted in red in S7 Table. Strong linkage: p-value chi-square test < 2.2*10E-16; linkage: p-value chi-square test < 0.01); this was not a problem with the BIITE algorithm. This shows that BIITE is successful in handling possible confounding due to linkage disequilibrium between genetic loci, a recurring problem that is not dealt with by RATE.

## Discussion

Determining the immunogenicity of peptide:HLA (pHLA) combinations is an important task when studying correlates of immunity in infection, which in turn may inform vaccine design. Different experimental and computational approaches have been used to establish this. The experimental approaches include assessing the binding strength of the pHLA complex, *in vitro* antigen presentation studies using CD4^+^ T cell clones, and HLA class II- transgenic mouse models to mimic pathogen peptide expression in a human host. Each of these methods has shortcomings. While binding (whether measured in binding assays or predicted) of a peptide and immunogenicity of that peptide are correlated, binding is insufficient to guarantee immunogenicity which entails other processes both in the antigen presentation pathways (such as cleavage of protein into smaller peptides and the loading of peptides onto HLA-II molecules) but also in the selection and expansion of the T cell clone(s) bearing the relevant TCR(s) *in vivo*. More direct assays of immunogenicity such as CD4^+^ T cell cloning from bulk cultures and assessment of peptide presentation using transfectants expressing individual HLA-II heterodimers are cumbersome and intractable for large-scale studies [19,20]. Transgenic mice are good *in vivo* models encompassing peptide processing and presenting pathways, but are labour-intensive to develop and maintain. Furthermore, the overlap between which peptides are immunogenic in humans and in mouse models is not perfect and seems to be highly dependent on the choice of the effector cell [21]. As the mouse model incorporates a single human HLA, it is also unsuitable to appreciate the effects of immunodominance, where different HLAs may present different peptides simultaneously to the host’s immune system [22].

ELISpot assays use *ex vivo* PBMCs from exposed hosts to test peptidic immunogenicity. In theory, this approach includes effects of peptide presentation pathway idiosyncrasies, binding affinities between peptide and HLA, and immunodominance. As a trade-off however, we lose the clarity of the experimental approaches where each HLA is studied independently, and it is difficult to assess which peptide:HLA combinations cause the observed ELISpot outcomes.

Here, we have presented a Bayesian framework to perform this analysis. We have shown its accuracy on both simulated and experimental data, and have determined the effect of sample size. While higher sample sizes (100–200 subjects) would have yielded better results, the analysis on a very limited dataset of 38 *Burkholderia*-sero-positive exposed subjects performed as expected (AUC = 71%) from simulated data. A caveat should be given regarding the composition of the data set: analysis on data sets with high rates of positive ELISpots suffer from poor identifiability. Nevertheless it is remarkable that with such a small dataset relatively high specificity and sensitivity could be achieved; while most algorithms for predicting immunogenicity or binding require training datasets with hundreds of data points per HLA allele, we typically have less than 10 data points per allele. Furthermore, unlike prediction algorithms, BIITE explicitly uses cohort data, which makes it suitable to interrogate differences in immunogenicity between patient groups responding differently to the same pathogen; this would be impossible for a binding prediction algorithm.

This tool is available online as the R package BIITE and is, in essence, not ELISpot or HLA class II-specific. In general, it can be used to attribute Boolean outcomes (such as therapeutic success or disease progression) to allelic variation on multiple genes. As an example, we have applied BIITE to an HLA class I ELISpot dataset (HIV-1). In general, to apply the algorithm to a new problem it is necessary to consider carefully the source of the priors if these are to be used; establish the length of the MH chain needed to obtain a representative sample of the posterior distribution; and to consider issues of sample size and collinearity in the dataset.

It also allows for the addition of prior information ad libitum: we can envisage a scenario where not only binding predictions (from NetMHCIIPan or other binding prediction algorithms) but also binding data and transgenic mouse data are included in the prior. Of course, conflicting binding information would result in different priors, and hence influence the outcome of the algorithm. However, the effect of the prior is dependent on sample size; in a suitably large dataset, the influence of the prior is outweighed by the likelihood factor. Alternatively, confidence in the prior can be expressed in the prior itself: the more ‘flat’ a prior, the less it will influence the posterior distribution.

In summary, we have developed a general approach which can be used for analysing complex biological data. Whether or not a peptide:HLA combination is immunogenic involves a number of interconnected immunological processes. To sidestep this complexity, reductionist experimental approaches are often taken, e.g. *in vitro* stimulation with APCs expressing a single HLA molecule; these approaches do not recapitulate a number of, potentially important, biological details. We have developed an approach that allows us to use the most pertinent but also most complex experimental data (ELISpot) and interrogate it to obtain accurate results.

## Supporting Information

S1 Fig Priors as used, depending on the predictions from NetMHCIIPan. A peptide:HLA combination is considered to be a binder if the predicted binding affinity is below 500nM; the prior for such a combination is the Beta distribution with mode 0.35 and SD 0.2. Peptide:HLA combination with predicted binding affinity above 500nM have the Beta distribution with mode 0.001 and SD 0.15 as prior.
(PNG)

S2 Fig A comparison of the odds ratios of having a positive ELISpot (i.e. number of positive ELISpots/number of negative ELISpots for that peptide) in the original *Burkholderia* dataset and the simulated data. (PNG)

S3 Fig Datasets with high ELISpot success rates are harder to analyse. (A) We ordered the *Burkholderia* peptides based on their ELISpot success rates and considered subsets of this dataset. We predict the AUCs to be negatively correlated with the success rate, i.e. the subsets to be ordered as shown in (B) (subset 8 predicted to have the lowest AUC, subset 1 the highest). This is indeed what we observe in (C).
(PNG)

S4 Fig Comparison to NetMHCIIPan. (A) For the *Burkholderia* data. (B) for the *Pseudomonas* data.
(PNG)

S5 Fig The algorithm avoids the following pitfalls. (A) Posterior modes are not correlated with the number of HLA carriers. (B) Posterior modes are not strongly correlated with the percentage of HLA carriers that produced a positive ELISpot. Data is taken from the analysis of the *Burkholderia* data set.
(PNG)

S1 Table Count and frequency of *DRB1* and *DQB1* alleles in the *Burkholderia* cohort. (DOCX)

S2 Table Count and frequency of *DRB1* and *DQB1* alleles in the *Pseudomonas* cohort. (DOCX)

S3 Table Discordant results between binding assay and transgenic mouse models in *Burkholderia* data set. (A) Peptide:MHC combinations which were found to be immunogenic in the transgenic mouse model but did not bind in the binding assay, together with their predicted binding IC_50_ from NetMHCIIPan, and the analysis of the posterior mode from our analysis. The Kullback-Leibler divergence of the marginal posterior with respect to the uniform distribution is also indicated. (B,C) Peptide:MHC combinations which were not immunogenic in the transgenic mouse assays but showed stable binding in the binding assay. Of these 34 combinations, 11 were predicted to be binding (IC_50_ < 500 nM) by NetMHCIIPan (B). Marginal posterior distributions from our model passed the Kullback-Leibler cut-off for 16/34 combinations (C). Between these two tables, only five were found in common (highlighted in matching colours).
(DOCX)

S4 Table Example of an input file. (CSV)

S5 Table Count and frequency of *HLA-A*, *-B* and *-C* alleles in the HIV-1 cohort. (DOCX)

S6 Table Analysis of the HIV-1 ELISpot dataset (BIITE). We analysed data from 32 peptides (first column). These 32 peptides were the ones studied in [10] and were chosen because they contain LANL HIV-1 A list epitopes that are restricted by HLAs which are abundant in the cohort; this HLA is in the column ‘Restricting HLA (RATE)’. We also include the p value reported by RATE for each peptide:HLA combination. In the next column, ‘Restricting HLA (BIITE)’, we list the HLAs identified using BIITE that appear to elicit CD8^+^ T cell responses in combination with the peptide. For these HLAs, we provide the number of carriers that presented a positive ELISpot response for the peptide in question and we count how in how many responding carriers of the HLA this HLA had the highest posterior mode (‘# Carriers with positive ELISpot and highest rank’). We also show the posterior mode attributed to the peptide:HLA combination. The last two columns document whether (and where) the peptide:HLA combination was found in the LANL database.
(XLSX)

S7 Table Analysis of the HIV-1 ELISpot dataset (RATE). Peptide:HLA combinations in the ‘concise results’ of RATE. Green highlighted alleles were detected by BIITE, while red highlighted alleles are probably not immunogenic in combination with the peptide, but appear to be so because of linkage disequilibrium.
(XLSX)

S8 Table Example of an output file (pep_1 from the simulated data). HLAs are ranked from highest to lowest posterior mode (‘Posterior Mode’) and we provide the number of carriers in the cohort (‘# Carriers’) and the number of carriers who responded to the peptide. We also show, per HLA, how many carriers have this HLA as their highest ranked HLA (‘# Carriers with highest rank’); and how many carriers who responded to the peptide have this HLA as their highest ranked HLA (‘# Carriers with positive ELISpot and highest rank’). This last column can be interpreted as the number of positive ELISpots that are to be attributed to the specific HLA.
(XLSX)

S1 Text Mathematical considerations. An explanation of the rationale and machinery behind BIITE aimed at readers who do not have a background in Bayesian analysis.
(DOCX)
