# Supplementary figures and images for: BIITE: A Tool to Determine HLA Class II Epitopes from T Cell ELISpot Data
Source: PLoS Comput Biol. 2016 Mar 8;12(3):e1004796. doi: 10.1371/journal.pcbi.1004796 (PMC4783075; doi:10.1371/journal.pcbi.1004796)

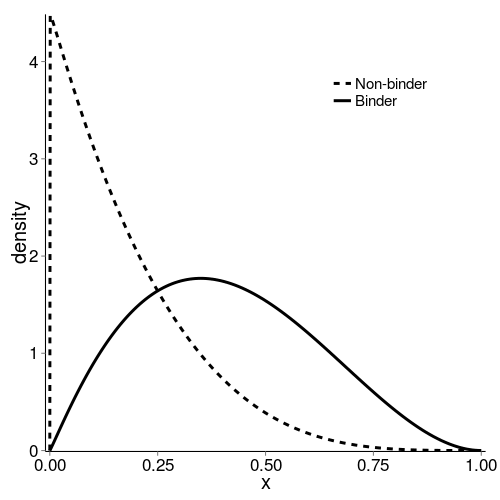

Supplement: S1 Fig — A peptide:HLA combination is considered to be a binder if the predicted binding affinity is below 500nM; the prior for such a combination is the Beta distribution with mode 0.35 and SD 0.2. Peptide:HLA combination with predicted binding affinity above 500nM have the Beta distribution with mode 0.001 and SD 0.15 as prior. (PNG) [file pcbi.1004796.s001.png]

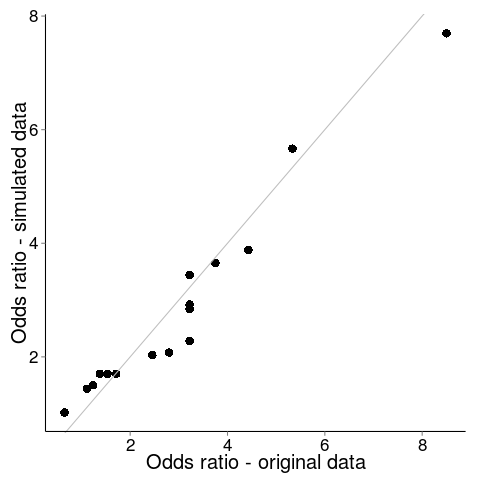

Supplement: S2 Fig — (PNG) [file pcbi.1004796.s002.png]

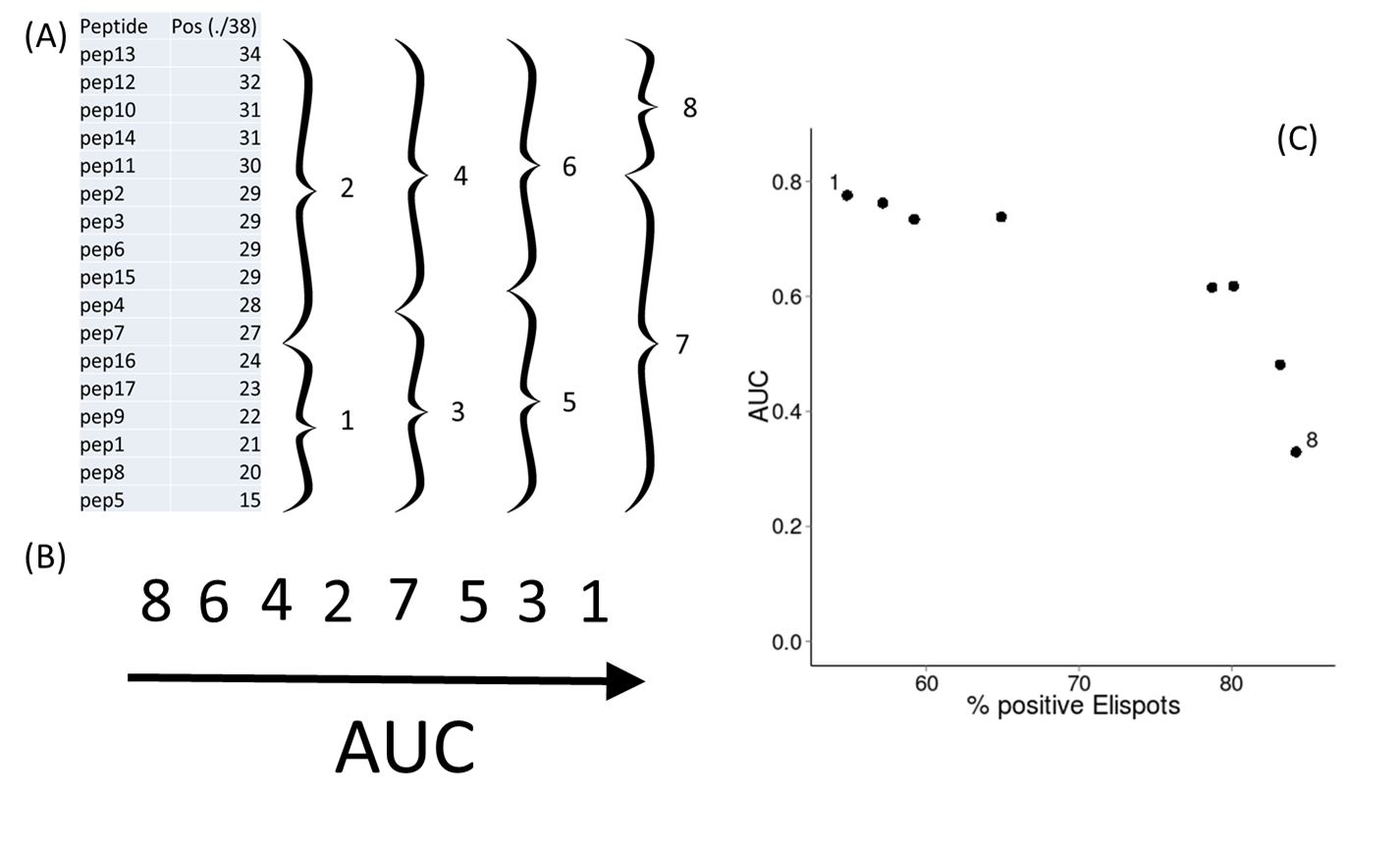

Supplement: S3 Fig — (A) We ordered the Burkholderia peptides based on their ELISpot success rates and considered subsets of this dataset. We predict the AUCs to be negatively correlated with the success rate, i.e. the subsets to be ordered as shown in (B) (subset 8 predicted to have the lowest AUC, subset 1 the highest). This is indeed what we observe in (C). (PNG) [file pcbi.1004796.s003.png]

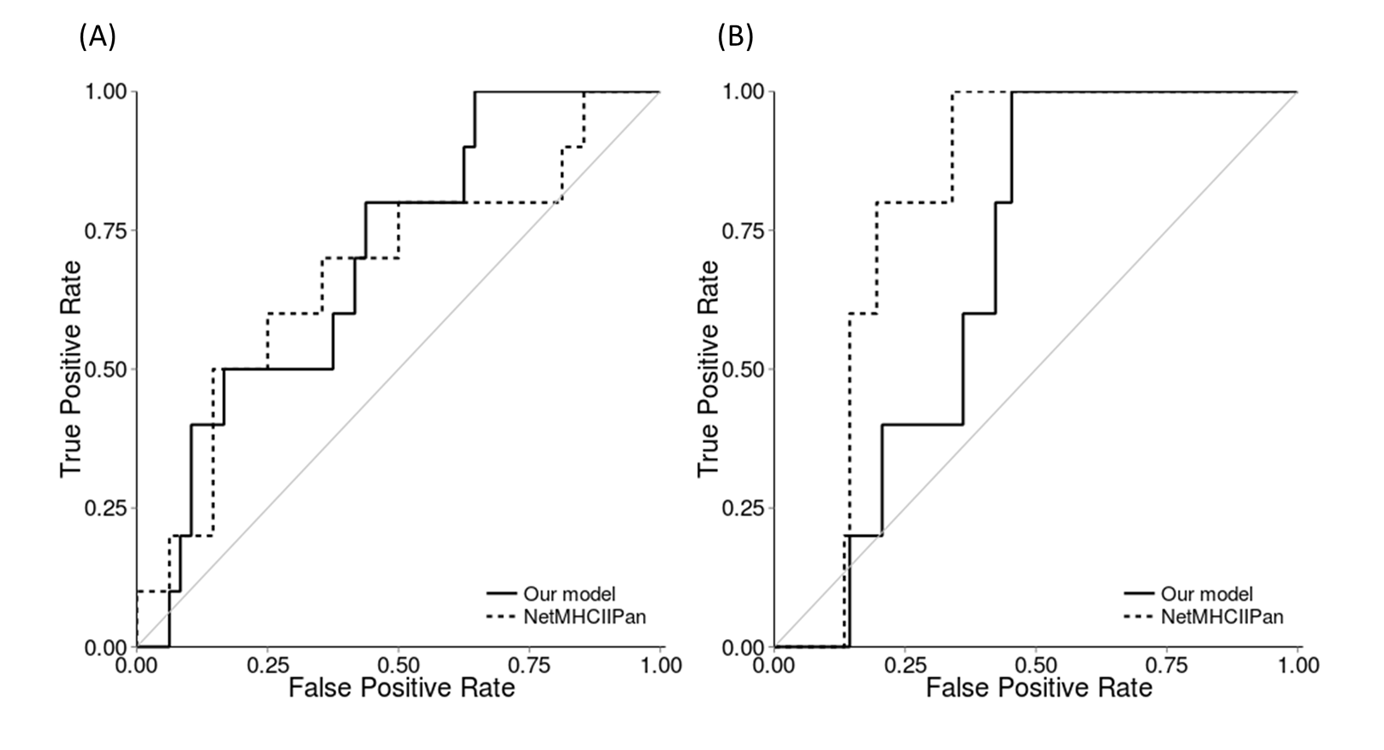

Supplement: S4 Fig — (A) For the Burkholderia data. (B) for the Pseudomonas data. (PNG) [file pcbi.1004796.s004.png]

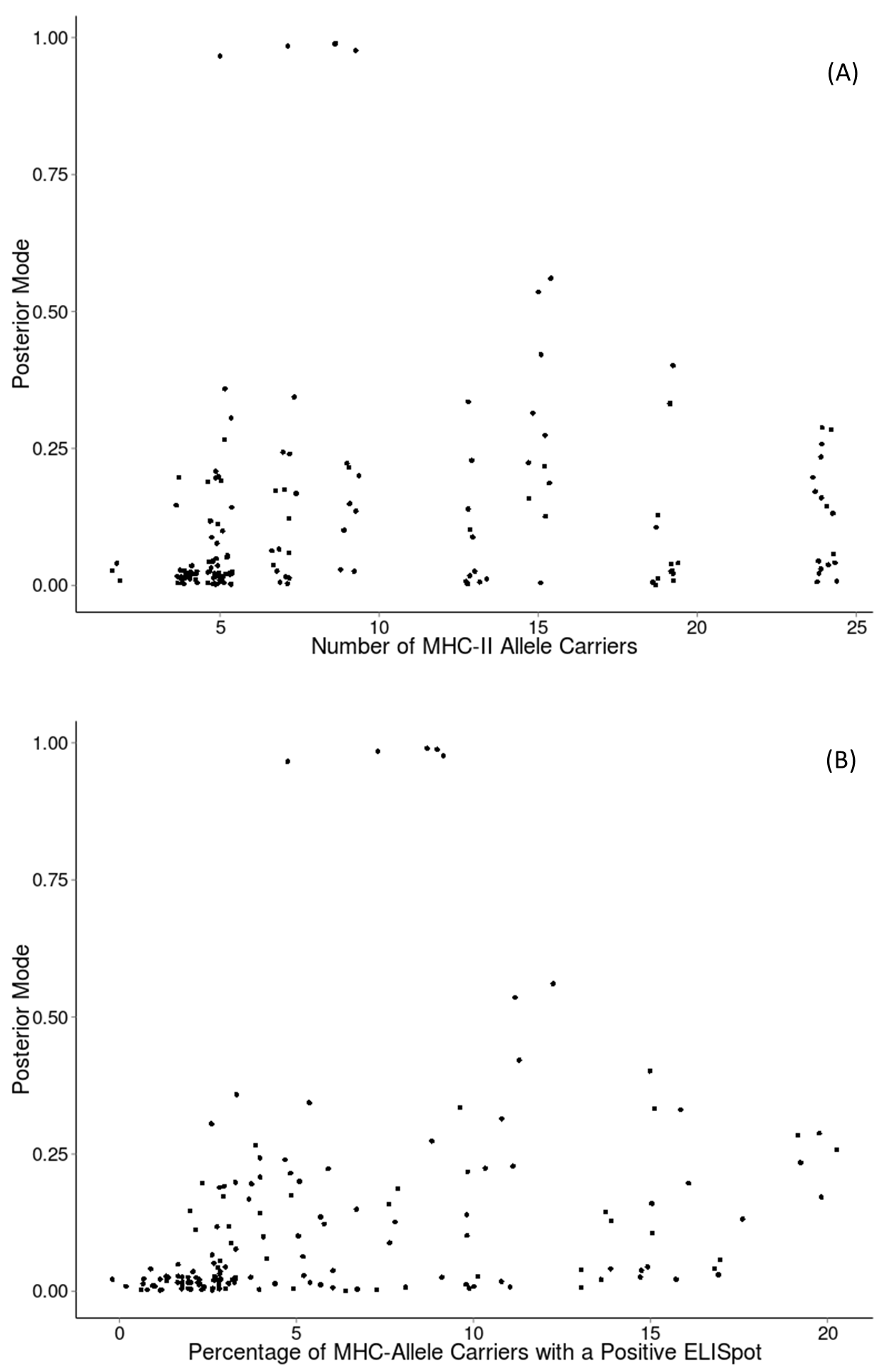

Supplement: S5 Fig — (A) Posterior modes are not correlated with the number of HLA carriers. (B) Posterior modes are not strongly correlated with the percentage of HLA carriers that produced a positive ELISpot. Data is taken from the analysis of the Burkholderia data set. (PNG) [file pcbi.1004796.s005.png]
