# Supplementary material for: BIITE: A Tool to Determine HLA Class II Epitopes from T Cell ELISpot Data
Source: PLoS Comput Biol. 2016 Mar 8;12(3):e1004796. doi: 10.1371/journal.pcbi.1004796 (PMC4783075; doi:10.1371/journal.pcbi.1004796)
Supplement: S1 Table — (DOCX) [file pcbi.1004796.s006.docx]

**S1 Table. Count and frequency of *DRB1* and *DQB1* alleles in the *Burkholderia* cohort.**

| HLA-II chain | Allele count | Allele frequency (%) |
| --- | --- | --- |
| *DRB1*01* | 2 | 2.63 |
| *DRB1*04* | 4 | 5.26 |
| *DRB1*07* | 5 | 6.58 |
| *DRB1*08* | 5 | 6.58 |
| *DRB1*09* | 8 | 10.53 |
| *DRB1*11* | 4 | 5.26 |
| *DRB1*12* | 17 | 22.37 |
| *DRB1*14* | 8 | 10.53 |
| *DRB1*15:01* | 9 | 11.84 |
| *DRB1*15:02* | 7 | 9.21 |
| *DRB1*16* | 5 | 6.58 |
| *DRB1*03* | 2 | 2.63 |
| *DQB1*02* | 5 | 6.58 |
| *DQB1*03* | 23 | 30.26 |
| *DQB1*04* | 2 | 2.63 |
| *DQB1*05* | 32 | 42.11 |
| *DQB1*06* | 14 | 18.42 |
