# Supplementary material for: BIITE: A Tool to Determine HLA Class II Epitopes from T Cell ELISpot Data
Source: PLoS Comput Biol. 2016 Mar 8;12(3):e1004796. doi: 10.1371/journal.pcbi.1004796 (PMC4783075; doi:10.1371/journal.pcbi.1004796)
Supplement: S2 Table — (DOCX) [file pcbi.1004796.s007.docx]

**S2 Table. Count and frequency of *DRB1* and *DQB1* alleles in the *Pseudomonas* cohort.**

| HLA-II chain | Allele Count | Allele  Frequency (%) |
| --- | --- | --- |
| *DRB1*01* | 18 | 15.52 |
| *DRB1*04* | 22 | 18.97 |
| *DRB1*07* | 12 | 10.34 |
| *DRB1*08* | 1 | 0.86 |
| *DRB1*09* | 2 | 1.72 |
| *DRB1*10* | 1 | 0.86 |
| *DRB1*11* | 7 | 6.03 |
| *DRB1*12* | 2 | 1.72 |
| *DRB1*13* | 8 | 6.9 |
| *DRB1*14* | 7 | 6.03 |
| *DRB1*15* | 24 | 20.69 |
| *DRB1*17* | 12 | 10.34 |
| *DQB1*02* | 21 | 18.1 |
| *DQB1*05* | 27 | 23.28 |
| *DQB1*06* | 32 | 27.59 |
| *DQB1*07* | 16 | 13.79 |
| *DQB1*08* | 15 | 12.93 |
| *DQB1*09* | 5 | 4.31 |
