# Supplementary material for: BIITE: A Tool to Determine HLA Class II Epitopes from T Cell ELISpot Data
Source: PLoS Comput Biol. 2016 Mar 8;12(3):e1004796. doi: 10.1371/journal.pcbi.1004796 (PMC4783075; doi:10.1371/journal.pcbi.1004796)
Supplement: S3 Table — (A) Peptide:MHC combinations which were found to be immunogenic in the transgenic mouse model but did not bind in the binding assay, together with their predicted binding IC50 from NetMHCIIPan, and the analysis of the posterior mode from our analysis. The Kullback-Leibler divergence of the marginal posterior with respect to the uniform distribution is also indicated. (B,C) Peptide:MHC combinations which were not immunogenic in the transgenic mouse assays but showed stable binding in the binding assay. Of these 34 combinations, 11 were predicted to be binding (IC50 < 500 nM) by NetMHCIIPan (B). Marginal posterior distributions from our model passed the Kullback-Leibler cut-off for 16/34 combinations (C). Between these two tables, only five were found in common (highlighted in matching colours). (DOCX) [file pcbi.1004796.s008.docx]

**S3 Table. Discordant results between binding assay and transgenic mouse models in *Burkholderia* data set.** (A) Peptide:MHC combinations which were found to be immunogenic in the transgenic mouse model but did not bind in the binding assay, together with their predicted binding IC_50_ from NetMHCIIPan, and the analysis of the posterior mode from our analysis. The Kullback-Leibler divergence of the marginal posterior with respect to the uniform distribution is also indicated. (B,C) Peptide:MHC combinations which were not immunogenic in the transgenic mouse assays but showed stable binding in the binding assay. Of these 34 combinations, 11 were predicted to be binding (IC_50_ < 500 nM) by netMHCPan (B). Marginal posterior distributions from our model passed the Kullback-Leibler cut-off for 16/34 combinations (C). Between those two tables, only five were found in common (highlighted in matching colours).

| (A)  Peptide number | HLA | NetMHCIIPan | Posterior Mode | DKL |
| --- | --- | --- | --- | --- |
| pep3 | DRB1*01 | 1050.16 | 0.931457 | 0.019513 |
| pep14 | DRB1*04 | **497.29** | 0.004264 | 0.141409 |
| pep2 | DRB1*15:01 | 953.47 | 0.516775 | 0.053087 |
| pep6 | DRB1*15:02 | 844.94 | 0.966079 | 0.240188 |
| pep17 | DRB1*15:02 | 4136.90 | **0.358841** | 0.192755 |

(B)

| Peptide  Number | HLA | NetMHCIIPAN | DKL |
| --- | --- | --- | --- |
| pep13 | DRB1*01 | 37.14 | 0.005695 |
| pep2 | DRB1*01 | 52.08 | 0.027483 |
| pep1 | DQB1*06 | 138.46 | 0.775687 |
| pep9 | DRB1*01 | 141.18 | 0.065565 |
| pep1 | DRB1*04 | 179.10 | 0.06673 |
| pep13 | DQB1*03 | 182.51 | 0.148588 |
| pep5 | DRB1*01 | 209.28 | 0.128466 |
| pep1 | DQB1*03 | 220.47 | 1.129249 |
| pep16 | DRB1*15:01 | 277.00 | 0.448482 |
| pep16 | DRB1*04 | 398.94 | 0.310061 |
| pep1 | DRB1*15:01 | 453.53 | 0.4561 |

(C)

| Peptide  Number | HLA | Posterior Mode | DKL |
| --- | --- | --- | --- |
| pep1 | DQB1*06 | 0.00396 | 0.775687 |
| pep16 | DRB1*04 | 0.004446 | 0.310061 |
| pep1 | DQB1*03 | 0.005689 | 1.129249 |
| pep5 | DQB1*06 | 0.006272 | 0.888444 |
| pep8 | DRB1*04 | 0.016085 | 0.29681 |
| pep7 | DQB1*06 | 0.025706 | 0.439444 |
| pep14 | DQB1*03 | 0.025878 | 0.598869 |
| pep8 | DQB1*03 | 0.027189 | 0.948019 |
| pep16 | DRB1*15:01 | 0.028677 | 0.448482 |
| pep16 | DQB1*03 | 0.039657 | 0.726847 |
| pep2 | DQB1*03 | 0.040863 | 0.555462 |
| pep10 | DQB1*03 | 0.106406 | 0.481225 |
| pep4 | DRB1*15:02 | 0.11798 | 0.366788 |
| pep4 | DQB1*06 | 0.139494 | 0.266345 |
| pep1 | DRB1*15:01 | 0.200311 | 0.4561 |
| pep7 | DQB1*03 | 0.401506 | 0.262062 |
