# Supplementary material for: BIITE: A Tool to Determine HLA Class II Epitopes from T Cell ELISpot Data
Source: PLoS Comput Biol. 2016 Mar 8;12(3):e1004796. doi: 10.1371/journal.pcbi.1004796 (PMC4783075; doi:10.1371/journal.pcbi.1004796)
Supplement: S5 Table — (DOCX) [file pcbi.1004796.s010.docx]

**S5 Table. Count and frequency of *HLA-A, -B*  and *-C* alleles in the HIV cohort.**

^a^ This includes: people who were not typed at the 4-digit level for *A*30* (and are potentially carrying *A*30:01* or *A*30:02*) and people who had been typed at the 4-digit level but were not carrying *A*30:01* or *A*30:02*. Similar for *B*58-rest* and *B*15-rest.*

| HLA | Allele count | Allele frequency |
| --- | --- | --- |
| *A*01* | 58 | 1.531959852 |
| *A*02* | 138 | 3.645007924 |
| *A*03* | 98 | 2.588483888 |
| *A*07* | 1 | 0.026413101 |
| *A*11* | 3 | 0.079239303 |
| *A*23* | 115 | 3.037506603 |
| *A*24* | 32 | 0.845219229 |
| *A*26* | 15 | 0.396196513 |
| *A*28* | 1 | 0.026413101 |
| *A*29* | 112 | 2.958267301 |
| *A*30:01* | 111 | 2.9318542 |
| *A*30:02* | 68 | 1.796090861 |
| *A*30-rest^a^* | 44 | 1.16217644 |
| *A*31* | 4 | 0.105652404 |
| *A*32* | 11 | 0.29054411 |
| *A*33* | 30 | 0.792393027 |
| *A*34* | 55 | 1.452720549 |
| *A*36* | 8 | 0.211304807 |
| *A*39* | 1 | 0.026413101 |
| *A*43* | 31 | 0.818806128 |
| *A*66* | 71 | 1.875330164 |
| *A*68* | 174 | 4.595879556 |
| *A*74* | 71 | 1.875330164 |
| *A*80* | 10 | 0.264131009 |
| *B*02* | 1 | 0.026413101 |
| *B*07* | 66 | 1.743264659 |
| *B*08* | 76 | 2.007395668 |
| *B*13* | 27 | 0.713153724 |
| *B*14* | 43 | 1.135763339 |
| *B*15:03* | 117 | 3.090332805 |
| *B*15:10* | 108 | 2.852614897 |
| *B*15-rest^a^* | 35 | 0.924458531 |
| *B*18* | 34 | 0.898045431 |
| *B*27* | 1 | 0.026413101 |
| *B*35* | 26 | 0.686740623 |
| *B*39* | 31 | 0.818806128 |
| *B*40* | 5 | 0.132065504 |
| *B*41* | 13 | 0.343370312 |
| *B*42* | 147 | 3.882725832 |
| *B*44* | 98 | 2.588483888 |
| *B*45* | 42 | 1.109350238 |
| *B*47* | 2 | 0.052826202 |
| *B*49* | 9 | 0.237717908 |
| *B*50* | 4 | 0.105652404 |
| *B*51* | 8 | 0.211304807 |
| *B*52* | 1 | 0.026413101 |
| *B*53* | 27 | 0.713153724 |
| *B*54* | 1 | 0.026413101 |
| *B*57* | 40 | 1.056524036 |
| *B*58:01* | 65 | 1.716851558 |
| *B*58:02* | 139 | 3.671421025 |
| *B*58-rest^a^* | 9 | 0.237717908 |
| *B*67* | 5 | 0.132065504 |
| *B*71* | 5 | 0.132065504 |
| *B*72* | 1 | 0.026413101 |
| *B*81* | 71 | 1.875330164 |
| *B*82* | 5 | 0.132065504 |
| *C*01* | 4 | 0.105652404 |
| *C*02* | 144 | 3.803486529 |
| *C*03* | 90 | 2.377179081 |
| *C*04* | 134 | 3.53935552 |
| *C*05* | 9 | 0.237717908 |
| *C*06* | 207 | 5.467511886 |
| *C*07* | 249 | 6.576862124 |
| *C*08* | 76 | 2.007395668 |
| *C*12* | 31 | 0.818806128 |
| *C*14* | 5 | 0.132065504 |
| *C*15* | 12 | 0.316957211 |
| *C*16* | 70 | 1.848917063 |
| *C*17* | 159 | 4.199683043 |
| *C*18* | 72 | 1.901743265 |
