# Supplementary material for: BIITE: A Tool to Determine HLA Class II Epitopes from T Cell ELISpot Data
Source: PLoS Comput Biol. 2016 Mar 8;12(3):e1004796. doi: 10.1371/journal.pcbi.1004796 (PMC4783075; doi:10.1371/journal.pcbi.1004796)
Supplement: S1 Text — An explanation of the rationale and machinery behind BIITE aimed at readers who do not have a background in Bayesian analysis. (DOCX) [file pcbi.1004796.s014.docx]

BIITE : Mathematical considerations

The aim of this supplementary text is to explain the concepts behind BIITE for people not familiar with Bayesian approaches. We will cover the following subjects:

1. Bayes’ theorem
2. The likelihood P(D|H)
3. Metropolis-Hastings algorithm

We will explain these concepts by using non-biological examples that appeal more to the intuition, before explaining how the concept is used in our method.

**Bayes’ theorem**

Bayes’ theorem is a mathematically exact way of describing how new data (D) changes our beliefs in a certain hypothesis (H) we might hold about reality. This is the theorem:

$$P\left( H | D \right)=\frac{P\left( D | H \right)P\left( H \right)}{C}.$$

An example

To illustrate this theorem we consider an example in which the police have arrested a suspect for a robbery. There are two hypotheses, *H*, about the reality of this robbery: either the suspect did it (*H_1_*) or the suspect didn’t do it (*H_2_*)*.* The detective has some belief about which of the two hypotheses is more likely to be true, e.g. P(*H_1_*) = 80% and P(*H_2_*) = 20%. These are the ­*prior* probabilities of each of the hypotheses being true. During interrogation, the suspect comes up with a strong alibi. This alibi, to the detective, is new data (*D*), and he now has to take this new information into account to update his beliefs in both hypotheses *H_1_* and *H_2_*. These new beliefs will be denoted by P(*H_1_*|*D*) and P(*H_2_*|*D*). Here, $P\left( X | Y \right)$ denotes a conditional probability: what is the probability that *X* is true if we know (or assume) that *Y* is true?

In order to get the updated probabilities, which we call *posterior* probabilities, we have to calculate P(*D*|*H*). Informally, this factor assesses how likely it is to see the data *D* that we have just observed, if we assume that the hypothesis *H* is true (we call this term the *likelihood*). How does this translate to our example? Under hypothesis *H_1_*, the suspect is the sought-after robber, and hence cannot have a strong alibi. Of course, there is always a slight chance that the police are being hoodwinked by the suspect and his alibi witness. We could therefore assign P(*D*|*H_1_*) = 5%. For the alternative hypothesis *H_2_*, not everyone who wasn’t robbing the bank will have a strong alibi for the time of the robbery: we assign P(*D*|*H_2_*) = 70%.

At this point, we can calculate the posterior probabilities by multiplying the prior and the likelihood of each of the hypotheses:

P(*H_1_*|*D*) = 80% x 5% / *C* = 4%/*C*,

P(*H_2_*|*D*) = 20% x 70% / *C* = 14%/*C*.

*C* is a normalising factor that is constant and so the probability of *H­_2_* is more than 3 times that of *H_1_*; we therefore favour hypothesis 2 over hypothesis 1.

BIITE and Bayes

In this subsection, we will explain how we use Bayes’ theorem in our method. First, we identify the two key players: the data *D* and the hypotheses *H*. After that, we will discuss the prior. The likelihood will be discussed in the next section.

The data *D* is the ELISpot dataset. Regarding the hypotheses, we want to infer HLA:peptide immunogenicity for a single peptide and multiple HLAs. Assume we are in the situation of Figure 1, where there are 2 HLAs in the population. We want to describe how immunogenic these HLAs are in combination with an (unnamed) peptide, using two numbers (one for each of the HLAs); we denote these by *E_1_* and *E_2_*. Roughly speaking, *E_i_* is the probability that a subject with HLA molecule i will produce a positive ELISpot when testing the peptide. Since *E_1_* and *E_2_* are probabilities, they take a value between 0 and 1. So we are looking for the pair (*E_1_*, *E_2_*) in the square [0,1]^2^ that best describes our ELISpot outcomes. In the general case, where we are considering *m* different HLAs in the population, hypotheses are no longer pairs of number in the (two-dimensional) square, but *m*-tuples (*E_1_*, *E_2_*,…, *E_m_*) in the *m*-dimensional hypercube [0,1]*^m^*.

Next we consider the prior information. As discussed above, the prior is a function that assigns a probability to each hypothesis under consideration. Because our hypothesis space is an *m*-dimensional hypercube, the prior is a function on this hypercube.

In the absence of prior information, we can use a *uniform* or *uninformative prior* in which no hypothesis is considered to be more or less likely than another. Each hypothesis *H* has a prior probability P(*H*) = 1.

We have also implemented the option of using binding data (either from NetMHCIIPan or from binding assays) in our method. For each HLA, the binding data either indicates that the HLA binds the peptide in question, or it doesn’t. If an HLA molecule binds the peptide, we assume the chance is higher that the HLA:peptide combination is immunogenic than if there is no binding. This can be seen on Supplementary Figure 1, where the prior for the ‘binding’ case is more right-skewed than the one for the non-binding case. To identify the prior probability that the HLA:peptide combination is immunogenic in *E*% of the cases, we evaluate the prior at *E/100*. In order to get the prior for a hypothesis (*E_1_*, *E_2_*, …, *E_m_*) – considering all HLAs at the same time instead of just one – we multiply the prior probabilities P(*E_i_*).

Of course, the user is free to choose set their own priors, based on literature reviews, binding data, or other experiments. Once a prior has been decided upon, we only need one more piece of information: the likelihood.

**The likelihood P(*D*|*H*)**

The likelihood describes how realistic our data *D* is under a given hypothesis *H*. In our method, the hypothesis describes how immunogenic HLA:peptide combinations are. Given the fact that we know a subject’s HLA genotype, and the hypothesis tells us how likely each HLA is to contribute to a positive ELISpot response, we can compute how likely the subject’s observed ELISpot response is. We start with an example describing a coin toss game; this will hopefully be an instructive analogy to the rationale behind the likelihood computation in BIITE, which we explain afterwards.

An example

Suppose we have *m* coins. They are all loaded, but in different ways: the first coin has probability *p_1_* of coming up heads, the second coin has probability *p_2_* of coming up heads, and so on. We play the following game: we will toss each of the *m* coins, and if one of them comes up heads, we win. What is the probability of winning this game? To calculate this, we look at the probability of losing this game. We start with the first coin. The probability of losing with the first coin is 1 – *p_1_*. Then we toss the second coin, which has a probability of losing of 1 – *p_2_*. So the probability of losing the game if there are only two coins is (1 – *p_1_*) (1 – *p_2_*) – we can multiply the probabilities here because the coin tosses are independent events. Similarly, with *m* coins, the probability of losing is equal to the product

(1 – *p_1_*) (1 – *p_2_*) … (1 – *p_m_*),

and the probability of winning this game with these particular *m* coins, is

1 - (1 – *p_1_*) (1 – *p_2_*) … (1 – *p_m_*),

because the sum of the probability of winning and the probability of losing has to be 1.

The likelihood in BIITE

In BIITE, each HLA is like a coin, which has a certain probability *E_i_* of eliciting a T cell response in a subject who has the *i*th HLA. Of course, no individual has all the HLAs in the population, so the probability of having a T cell response to the peptide (‘winning’ the coin toss game), is only dependent on the HLAs that person has – in the coin game analogy, not everyone has all the coins. Suppose our subject has a negative ELISpot outcome. The probability of this happening, under the hypothesis *H* = (*E_1_*, *E_2_*, …, *E_m_*) is equal to

$$\left( 1-E_{1} \right)^{n_{1}} \left( 1-E_{2} \right)^{n_{2}}\ldots\left( 1-E_{m} \right)^{n_{m}}$$

where *n_1_* = 2 if the subject is homozygous for the first HLA, 1 if the person is heterozygous for the first HLA, and 0 else – and similarly for the other exponents. This way, we only take the HLAs into account that are actually found in the subject (since *x^0^*= 1 for all *x*). Similarly, if the subject has a positive ELISpot, the probability of this happening is the complement of the product above.

Hence, for each person, given their HLA genotype and their ELISpot outcome, we can calculate how likely this outcome was under the hypothesis at hand. To get an overall assessment of how likely the ELISpot outcomes are over the whole cohort, we multiply the likelihoods of all the subjects.

At this point, we have defined the prior probabilities for all the hypotheses, and we know how to calculate the likelihoods. This means we have almost all the information on the right hand side of the equation in Bayes’ theorem. It is hard to calculate the normalizing constant *C*, which we would need if we wanted to exactly determine the posterior probabilities for each hypothesis. However, since C is a constant, and since we are only interested in finding the hypothesis that best explains our ELISpot data, knowing *C* is not essential to solve the question of HLA:peptide immunogenicity.

**The Metropolis-Hastings (MH) algorithm**

We have all the information to calculate the posterior distribution P(*H*|*D*) up to a constant *C*. We are interested in finding out which hypotheses *H* in the hypercube [0,1]*^m^*  have a high posterior probability. In order to do so, we will sample hypotheses: this means we will be picking a (high) number of hypotheses in a way that hypotheses with higher posterior probability are more likely to be sampled. In the paper, we sampled until we had 100,000 hypotheses in our sample. Based on these samples, we computed the posterior mode of each HLA:peptide immunogenicity, which was then used to evaluate the performance of BIITE.

Here, we show how the MH algorithm works on a simpler example, where we are sampling numbers according to the normal distribution. So, instead of sampling from an *m*-dimensional hypercube, we are now sampling real numbers. Let *f* denote the probability density function of the normal distribution. In the figure below, *f* is depicted in black.

We will start with a random real number, *x_0_*. Next, we pick a number close to this initial value, *y*. We compute the acceptance ratio *f*(*y*)/ *f*(*x_0_*). If the acceptance ratio (AR) is greater or equal to 1, we add *y* to the sample. If the AR < 1, we will add either *y* to the sample, with probability equal to the acceptance ratio, or *x_0_*. To decide which of the two values is added to the sample, we take a random number between 0 and 1, *u*. If *u* is smaller than the AR, *y* is added to the sample; otherwise, we add (another copy of) *x_0_* to the sample. In any case, we have now found the second sampling value, *x_1_* and can repeat the process: find a value *y* close to *x_1_,* compute the acceptance ratio *f*(*y*)/ *f*(*x_1_*), and decide whether *y* or *x_1_* is added to the sample. This is repeated several thousands of times (depending on the problem).

In the series of pictures below, we use the MH algorithm to sample from the normal distribution (outlined in black). The blue area represents the sample distribution at different times during the sampling process. In the beginning, we have just a few samples, and they do not represent the normal distribution well. But if we keep adding more values to our sample, we see that the sample distribution is very similar to that of a normal distribution (in black).


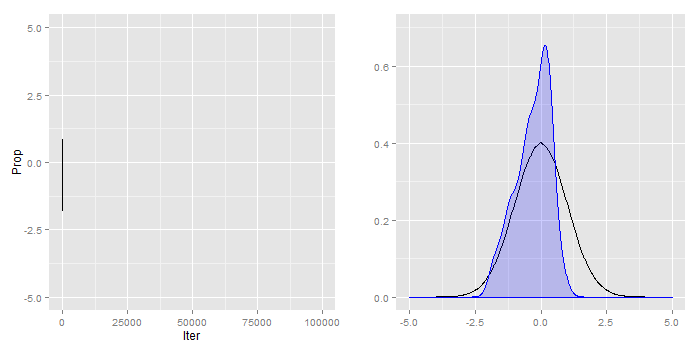

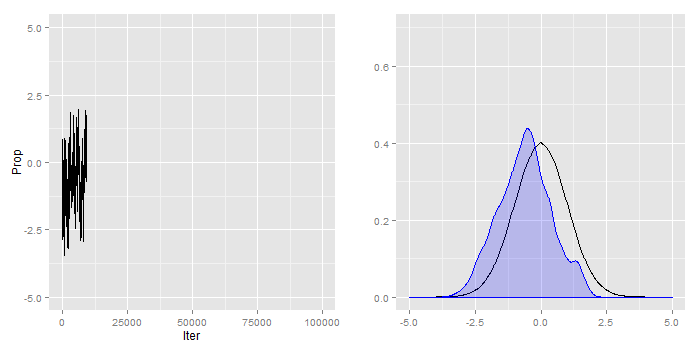

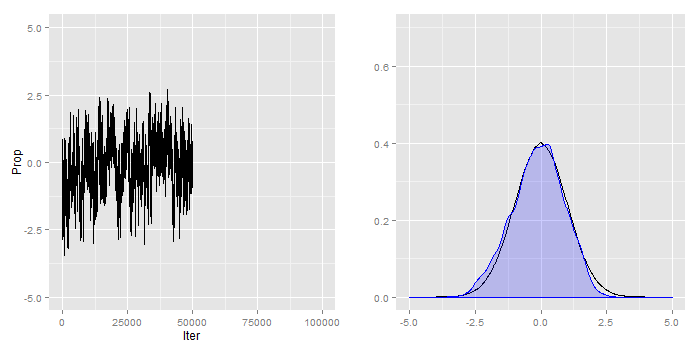


More samples added
